# Patient-reported outcomes after one-stage neourethral reconstruction in transmen with phalloplasty-associated strictures and fistulas

**DOI:** 10.1007/s00345-024-05246-0

**Published:** 2024-09-30

**Authors:** Victor M. Schuettfort, Rebecca R. Graf, Malte W. Vetterlein, Tim A. Ludwig, Philipp Gild, Phillip Marks, Armin Soave, Roland Dahlem, Margit Fisch, Silke Riechardt

**Affiliations:** 1 Department of Urology, University Medical Center Hamburg-Eppendorf, Martinistraße 52, 20246 Hamburg, Germany; 2 Department of Urology, Hospital Itzehoe, Itzehoe, Germany

**Keywords:** Transgender, Urethral reconstruction, Fistula, Stricture, Trans men, Gender-affirming surgery

## Abstract

**Introduction:**

Urethral strictures and fistulas arising after gender-affirming surgery in transmen require meticulous management strategies. This study evaluates the safety and efficacy of urethral reconstruction and patient satisfaction post-surgery.

**Methods:**

A retrospective analysis examined peri- and postoperative data from transmen undergoing urethral reconstruction for urethral fistula and/or strictures at the distal urethral anastomosis between December 2017 and April 2023. Follow-up involved clinical examinations, uroflowmetry, and voiding cystourethrography. Patient satisfaction and quality of life were assessed using USS PROM and ICIQ-S questionnaires.

**Results:**

Among 25 patients, 88% (*n* = 23) had urethral fistulas, and 48% (*n* = 12) had urethral strictures. 41% of fistula patients also had strictures, while 75% of stricture patients had concurrent fistulas. Previous surgeries for fistula or stricture repair were noted in 26% of cases. Techniques for stricture included modified flap (50%), buccal oral mucosal grafting (33%), and primary anastomosis (17%). Post-operative urethrogram revealed urethral strictures in 15% (*n* = 3) and urinary extravasation in an equal number. Postoperative uroflow parameters showed improvement (Qmax 18 ml/s, Qave 7.9 ml, time 37 s, volume 332 ml). Perioperative complications were low (*n* = 6, 24%), all grade one (Clavien-Dindo). Follow-up revealed that 33% required another surgical intervention. The mean six-item LUTS score was 6.7 (SD 3.9). Mean ICIQ-S overall satisfaction score was 8.6 (SD 1.6) and outcome score was 20 (SD 2.8).

**Discussion:**

Our study found a significant recurrence rate of urethral strictures and fistulas post-surgery. Despite this, patient satisfaction remains high and complications are generally low-grade, highlighting the importance of expert surgical intervention.

## Introduction

Gender-affirming surgery plays a pivotal role in the holistic care of transgender individuals striving to align their physical appearance with their gender identity. The number of these surgeries performed has experienced unprecedented growth during the last years [1], with trans men being more likely to undergo surgery than transwomen [2]. To mitigate gender dysphoria, transgender men transition entails a series of surgical interventions, including chest - and genital reconstruction, with phalloplasty making up < 5% of those surgeries in the United States [3]. Among the variety of gender-affirming surgeries, an integral facet of transgender men transition lies in managing urethral complications after phalloplasty, notably urethral fistulas and strictures. These complications, occurring with high frequency, profoundly impact patient satisfaction and quality of life [4, 5].

Despite advancements in surgical techniques and enhanced patient care, the incidence of urethral complications such as fistulas or strictures in transgender patients undergoing transgender men transition remains relatively high [1, 2]. The literature describes around 14–60% of patients undergoing phalloplasty develop urethral strictures and/or urethral fistulas post-operatively, with 94–96% leading to the need of revision surgeries [3–7]. Strictures are most frequently localized at the distal urethral anastomosis between the fixed and pendulous neo-urethra [4, 8]. The complexity and elevated risk of complications in this patient cohort are attributable to several factors, including prior surgical scarring, hormonal influences on tissue healing as well as challenges related to tissue availability and vascularity [7, 9]. Consequently, multiple surgical techniques have been explored to improve surgical results for urethral reconstruction. However, existing studies are characterized by small sample sizes, retrospective nature, heterogeneous patient cohorts and high recurrence rates [8, 10]. Thus, the absence of standardized guidelines and an evidence-based approach to surgical management persists. This lack of high-quality data magnifies the need for comprehensive research evaluating diverse surgical techniques and objectifiable patient-reported outcomes. There is an urgent demand to critically assess available evidence, compare surgical approaches, and identify the most effective techniques, to ultimately better patient satisfaction.

Thus, the aim of our study is assessing the outcomes of reconstructive surgery as well as the resulting patient satisfaction in a homogenous patient cohort. The data was gained from the database of our high-volume reconstructive referral center. Apart from surgical outcomes and functional results, patient-reported outcomes were collected and analyzed for the first time. This study aims to emphasize and enhance the guidance for perioperative management of a patient population that is often overlooked.

## Patients and methods

In accordance with our institutional review board’s approval (PV4123), perioperative and follow-up data of all transgender men undergoing urethral reconstruction for either urethral fistula and/or stricture of the distal urethral anastomosis at our multidisciplinary transgender center between December 2017 and April 2023 were retrospectively collected. Exclusion criteria for this analysis were individuals below 18 years old, non-anastomotic strictures and anterolateral thigh flap phalloplasty.

### Evaluation, surgical procedure, and perioperative management

We evaluated preoperative clinical characteristics, including age, American Society of Anesthesiologists (ASA) score, body mass index (BMI), history of colpectomy, implantation of penile or testicular prostheses, previous surgical urethral interventions as well as other comorbidities. Preoperative stricture evaluation involved combined retrograde urethrography and voiding cystourethrography to determine stricture location and length. All procedures were performed at least three months after phalloplasty or the most recent transurethral intervention. Surgical characteristics such as operative time were recorded. Additional preoperative diagnostics included uroflowmetry and a urine culture to rule out an acute infection. In cases of urinary retention, a suprapubic tube was placed before surgery. 30-day complications were assessed using the Clavien-Dindo classification.

### Surgical procedure

All procedures were conducted by two experienced high-volume reconstructive surgeons (S.R. and R.D). Perioperative management followed a standardized institutional protocol in line with World Professional Association for Transgender Health (WPATH) recommendations. Hormonal therapy was not interrupted. An 18-French Foley catheter was placed in all cases and in select cases, also a suprapubic tube at the discretion of the surgeon. A single intravenous dose of cefuroxime 1.5 g was routinely administered at the beginning of the operation. For urethral strictures, urethroplasty involved either excision and primary anastomosis (EPA; resection of obliterated urethra and re-anastomosis), an augmentation via a buccal mucosal graft or a modified flap technique. The latter technique is a modified primary anastomosis which also utilizes tissue located proximal to the stricture for flap creation to cover the urethral defect. Fistulas were managed either through simple primary closure followed by multi-layered closure, a flap technique, or excision via a YV plasty. The choice of operative technique was primarily based on intraoperative findings and patient history, and it was at the surgeon’s discretion. Important intraoperative findings influencing the choice of operative technique included the availability of sufficient proximal urethral tissue for modified end-to-end anastomosis, the extent of scar tissue, the availability of grafting tissue, and the patient’s history of previous reconstruction techniques such as buccal mucosal grafting.

### Follow-up, definition of stricture recurrence and patient-reported outcome measurement

All patients were discharged from the hospital with Foley catheter and/or suprapubic tube in place. Postoperatively, all patients attended our outpatient clinic for clinical check-ups, voiding cystourethrography, and uroflowmetry. Generally, 21 days postoperatively, a voiding cystourethrography was performed after catheter removal. Subsequent follow-ups were typically overseen by local urologists after a successful catheter removal.

The follow-up adhered to our institutional protocol, involving clinical examination, uroflow and voiding cystourethrography to objectively assess functional outcomes [11]. Chart reviews were conducted for readmissions and revisits to our institution.

Stricture recurrence was defined as symptomatic need for any instrumentation (dilation, endoscopic or open reconstructive surgery) or evidence of a stricture on imaging [12]. Fistulas were defined as clinical diagnosis of urethrocutaneous fistula or evidence of a fistula on imaging. The recurrence was analyzed via chart review, a telephone survey, and the standardized questionnaires sent out by mail.

### Patient-reported outcomes

The Patient-Reported Outcome Measures (PROMs) were sent out at the time of follow-up. Two different standardized German questionnaires have been used to gather information for the follow-up [13, 14]. PROM scoring incorporated a descriptive analyses of functional outcomes represented by the Urethral Stricture Surgery (USS) PROM and International Consultation on Incontinence Questionnaire Satisfaction (ICIQ-S) including the six-item lower urinary tract symptoms (LUTS) score (reference range 0–24) with a lower score corresponding to less bothersome symptoms, ICIQ-S outcome score (reference range 0–24), and ICIQ overall satisfaction with surgery item score (reference range 0–10), with higher scores corresponding to greater treatment satisfaction.

### Statistical analyses

Descriptive analyses summarized baseline, treatment, and surgical characteristics. Categorical variables were reported using frequencies and proportions, while continuous variables were represented using mean and standard deviation (SD) or median and interquartile range (IQR) based on normal or non-normal distribution, determined by Fisher’s exact test or Wilcoxon rank-sum test as appropriate. All statistical analyses were performed using R (Version 4.0.3, Vienna, Austria, 2020).

## Results

Urethral reconstruction for either stricture or fistula was performed in a total of 25 included patients between 12/2017 and 04/2023. 23 patients (88%) had urethral fistulas and 12 (48%) suffered from urethral strictures. Nine (41%) of all fistula patients also suffered from strictures, while 75% of all stricture patients suffered from urethral fistulas. Six patients (26%) underwent previous surgery for either fistula repair or stricture repair. Median Qmax of the preoperative uroflowmetry was low in both groups, despite only minimal residual urine. Median patient age was 30 years (IQR 26–35) and median BMI was 27 (IQR 22–32). All patients had an ASA score of one or two, indicating no or mild systemic disease respectively. Nearly half of patients were active smokers (48%). The most frequent comorbidities were depression (32%), asthma (20%), hypothyroidism and hypertension (each 16%). Further patient characteristics are displayed in Table 1.

**Table 1:** Baseline, treatment, and surgical characteristics of transmen who underwent urethral reconstruction for fistula or stricture after gender genital affirming surgery

	Overall	Fistula patients	Stricture patients
n	25 (100%)	22 (88%)	12 (48%)
Age	30.0 (26.0, 35.0)	30.5 (26.2, 35.0)	31.5 (26.8, 35.8)
BMI	27.0 (22.3, 31.9)	26.2 (22.2, 31.7)	30.4 (27.1, 34.7)
Hypothyreosis	4 (16%)	3 (14%)	4 (33%)
Asthma	5 (20%)	5 (23%)	3 (25%)
Hypertension	4 (16%)	3 (14%)	2 (17%)
Depression	8 (32%)	8 (36%)	3 (25%)
Smoking	11 (44%)	10 (45%)	4 (33%)
ASA	2.00 (2.00, 2.00)	2.00 (2.00, 2.00)	2.00 (2.00, 2.00)
Number of previous urethral reconstructions			
• none	16 (73%)	15 (75%)	5 (56%)
• one	3 (14%)	3 (15%)	3 (33%)
• Two or more	3 (14%)	2 (10%)	1 (11%)
• Unknown	3	2	3
Diverticulum	3 (12%)	3 (14%)	3 (25%)
Testis prothesis implanted			
• no	13 (52%)	11 (50%)	6 (50%)
• yes	4 (16%)	4 (18%)	2 (17%)
• implanted later	8 (32%)	7 (32%)	4 (33%)
Penile prothesis implanted			
• no	13 (52%)	11 (50%)	4 (33%)
• yes	2 (8.0%)	2 (9.1%)	1 (8.3%)
• previously explanted	9 (36%)	8 (36%)	6 (50%)
• implanted later	1 (4.0%)	1 (4.5%)	1 (8.3%)
Preoperative Uroflow - Qmax (ml/s)	10 (4, 30) (*n* = 13)	16 (7, 33) *n* = 10	4 (4, 10) *n* = 7
Preoperative Uroflow – Qave (ml/s)	5.4 (2.5, 12.0) (*n* = 13)	8.8 (3.4, 12.8) *n* = 10	2.9 (2.2, 5.4) *n* = 7
Preoperative Uroflow -Volume (ml)	242 (192, 397) *n* = 13)	280 (165, 462) *n* = 10	215 (178, 242) *n* = 7
Preoperative Uroflow - Flow time (seconds)	54 (36, 96) *n* = 12	41 (35, 71) *n* = 9	71 (46, 110) *n* = 7
Preoperative Uroflow residual urine (ml)	20 (0, 125) *n* = 10	25 (0, 130) *n* = 8	12 (4, 51) *n* = 5

**Table 2:** Postoperative outcome of transmen who underwent urethral reconstruction for fistula or stricture after gender genital affirming surgery

Characteristic	Overall	Fistula patients	Stricture patients
n	*N* = 25	yes, *N* = 22	yes, *N* = 12
Median Follow up in Months (IQR)	37 (16–45)	36 (18–43)	38 (22–45)
Mean Postoperative Uroflow - Qmax (SD) ml/s	21 (10)	21 (11)	21 (12)
• Unknown	5	5	1
• Change in %	110	31.25	425
Mean Postoperative Uroflow - Q-average (SD) ml/s	9.6 (4.7)	9.4 (4.6)	9.3 (5.7)
• Unknown	4	4	1
• Change in %	77.7	6.81	220.7
Mean Postoperative Uroflow - Flow time (SD) seconds	48 (38)	52 (41)	55 (50)
• Unknown	5	5	1
• Change in %	-11.1	+ 26.8	-22.5
Mean Postoperative Uroflow - Volume (SD) ml	319 (144)	340 (145)	304 (137)
• Unknown	4	4	1
• Change in %	31.8	21.4	41.4
Mean Postoperative Uroflow - Residual urine (SD) ml	24 (23)	24 (22)	29 (28)
• Unknown	6	5	3
• Change in %	20.0	-4.0	141.6
“Are you bothered by drippling”			
• Yes, a lot	2 (15%)	2 (18%)	1 (12%)
• Yes, to some degree	6 (46%)	4 (36%)	4 (50%)
• No, not really	4 (31%)	4 (36%)	2 (25%)
• No, not at all	1 (7.7%)	1 (9.1%)	1 (12%)
“Can this be improved by milking of the urethra?"			
• Yes, to some degree	6 (46%)	5 (45%)	3 (38%)
• Yes, completely	2 (15%)	2 (18%)	2 (25%)
• No, not really	2 (15%)	2 (18%)	1 (12%)
• No, not at all	3 (23%)	2 (18%)	2 (25%)
**USS-Prom**	**Frequencies (proportions**,** %)**		
Question 1: Is there a delay before you start to urinate?			
• Never	5 (45%)	5 (56%)	3 (50%)
• Occasionally	2 (18%)	1 (11%)	2 (33%)
• Sometimes	3 (27%)	2 (22%)	1 (17%)
• Most of the time	1 (9.1%)	1 (11%)	0 (0%)
• All the time	0 (0%)	0 (0%)	0 (0%)
Question 2: Would you say that the strength of your urinary stream is…			
• Normal	6 (55%)	5 (56%)	4 (67%)
• Occasionally reduced	3 (27%)	2 (22%)	2 (33%)
• Sometimes reduced	2 (18%)	2 (22%)	0 (0%)
• Reduced most of the time	0 (0%)	0 (0%)	0 (0%)
• Reduced all of the time	0 (0%)	0 (0%)	0 (0%)
Question 3: Do you have to strain to continue urinating?			
• Never	4 (36%)	3 (33%)	4 (67%)
• Occasionally	4 (36%)	3 (33%)	2 (33%)
• Sometimes	3 (27%)	3 (33%)	0 (0%)
• Most of the time	0 (0%)	0 (0%)	0 (0%)
• All the time	0 (0%)	0 (0%)	0 (0%)
Question 4: Do you stop and start more than once while you urinate?			
• Never	7 (64%)	5 (56%)	5 (83%)
• Occasionally	3 (27%)	3 (33%)	1 (17%)
• Sometimes	1 (9.1%)	1 (11%)	0 (0%)
• Most of the time	0 (0%)	0 (0%)	0 (0%)
• All the time	0 (0%)	0 (0%)	0 (0%)
Question 5: How often do you feel your bladder has not emptied properly after you have urinated?			
• Never	5 (45%)	4 (44%)	4 (67%)
• Occasionally	4 (36%)	3 (33%)	2 (33%)
• Sometimes	2 (18%)	2 (22%)	0 (0%)
• Most of the time	0 (0%)	0 (0%)	0 (0%)
• All the time	0 (0%)	0 (0%)	0 (0%)
Question 6: How often have you had a slight wetting of your pants a few minutes after you had finished urinating and had dressed yourself?			
• Occasionally	1 (12%)	1 (17%)	0 (0%)
• Sometimes	3 (38%)	2 (33%)	2 (50%)
• Most of the time	2 (25%)	2 (33%)	0 (0%)
• All the time	2 (25%)	1 (17%)	2 (50%)
• unknown	3	3	2
Peeling’s voiding picture			
• 1	1 (9.1%)	0 (0%)	1 (17%)
• 2	9 (82%)	8 (89%)	4 (67%)
• 3	1 (9.1%)	1 (11%)	1 (17%)
**ICIQ-Satisfaction**	**Frequencies (proportions**,** %)**		
Question 1 - How would you rate the outcome of your surgery?			
• very successful	7 (64%)	6 (67%)	4 (67%)
• somewhat succesful	4 (36%)	3 (33%)	2 (33%)
• Neither successful nor unsuccessful	0	0	0
• A little unsuccessful	0	0	0
• Very unsuccessful	0	0	0
Question 2 - Compared to how you felt before your surgery, how is your condition now?			
• much better	9 (82%)	7 (78%)	6 (100%)
• a bit better	1 (9.1%)	1 (11%)	0 (0%)
• about the same	1 (9.1%)	1 (11%)	0 (0%)
• A bit worse	0	0	0
• Much worse	0	0	0
Question 3 - Would you say you have been able to return to a ‘normal life’ after your surgery?			
• Strongly disagree	0	0	0
• Disagree	0	0	0
• agree	5 (45%)	4 (44%)	3 (50%)
• strongly agree	6 (55%)	5 (56%)	3 (50%)
Question 4 - If you were in the same situation again, would you still have the surgery?			
• yes, definitely	10 (91%)	8 (89%)	5 (83%)
• Yes, probably	0	0	0
• not sure	1 (9.1%)	1 (11%)	1 (17%)
• No, probably not	0	0	0
• No, definitely not	0	0	0
Question 5 - Would you recommend this surgery to friends or relatives with similar problems?			
• yes, definitely	9 (82%)	7 (78%)	5 (83%)
• yes, probably	1 (9.1%)	1 (11%)	0 (0%)
• not sure	1 (9.1%)	1 (11%)	1 (17%)
• No, probably not	0	0	0
• No, definitely not	0	0	0
Question 6 - If you had to spend the rest of your life with your symptoms as they are now, how would you feel?			
• perfectly happy	1 (9.1%)	1 (11%)	0 (0%)
• somewhat happy	3 (27%)	2 (22%)	1 (17%)
• Mixed feeling	0	0	0
• somewhat unhappy	1 (9.1%)	1 (11%)	1 (17%)
• desperate	6 (55%)	5 (56%)	4 (67%)
Question 7 - How prepared did you feel for the surgery?			
• completely prepared	6 (55%)	5 (56%)	3 (50%)
• somewhat prepared	4 (36%)	3 (33%)	3 (50%)
• somewhat unprepared	1 (9.1%)	1 (11%)	0 (0%)
• completely unprepared	0	0	0
Question 8 - How satisfied were you with any explanation about your surgery?			
• Very satisfied	6 (55%)	5 (56%)	4 (67%)
• Quite satisfied	3 (27%)	3 (33%)	1 (17%)
• Quite dissatisfied	1 (9.1%)	1 (11%)	0 (0%)
• Very dissatisfied	0	0	0
• I did not receive any information	1 (9.1%)	0 (0%)	1 (17%)
Question 9a - After the surgery, how much pain did you experience?			
• None	1 (9.1%)	1 (11%)	0 (0%)
• A little	8 (73%)	6 (67%)	4 (67%)
• A lot	1 (9.1%)	1 (11%)	1 (17%)
• Severy pain	1 (9.1%)	1 (11%)	1 (17%)
Question 9b - How much did this bother you? (Score between 0 (not at all) and 10 (a great deal)) (SD)	3.62 (2.75)	3.55 (2.88)	3.62 (2.56)
Question 10 - How satisfied were you with any treatment you had for pain?			
• I did not require pain treatment	0	0	0
• Very satisfied	0	0	0
• Quite satisfied	7 (64%)	6 (67%)	5 (83%)
• Quite dissatisfied	4 (36%)	3 (33%)	1 (17%)
• Very dissatisfied	0	0	0
Question 11a - How much pain are you experiencing now?			
• None	9 (82%)	7 (78%)	5 (83%)
• A little	2 (18%)	2 (22%)	1 (17%)
• A lot	0	0	0
• Severy pain	0	0	0
Question 11b - How much does this bother you? (Score between 0 (not at all) and 10 (a great deal))(SD)	0.46 (0.97)	0.55 (1.04)	0.38 (1.06)
Question 12 - Were there any complications or side effects with your surgery?			
• No complications	4 (36%)	3 (33%)	3 (50%)
• Yes, minor complications	7 (64%)	6 (67%)	3 (50%)
• Yes, major complications	0	0	0
Question 13 Overall, has the result of your surgery been…?			
• Better than expected	6 (55%)	5 (56%)	4 (67%)
• different than expected	3 (27%)	3 (33%)	0 (0%)
• just as expected	2 (18%)	1 (11%)	2 (33%)
• worse than expected	0	0	0
ICIQS14 - Overall, how satisfied were you with your surgery? ( a number between 0 (not satisfied) and 10 (very satisfied)) (SD)	8.62 (1.61)	8.64 (1.63)	8.62 (1.85)

Median operative time was 55 min (IQR 38–75). Of 12 patients with a stricture, the flap technique was used in six patients (50%), while buccal mucosal grafting was performed in four patients (33%) and EPA was resorted to in two patients (17%). In 23 patients with a fistula, the flap technique was also the most frequently used option in seven patients (50%), while a primary closure was performed in five (36%) and a YV plasty was employed in two patients (14%). In most patients, a colpectomy had either been previously performed (24%) or was performed simultaneously during the surgery (40%). Median catheter placement was 21 days and, in most cases, a suprapubic catheter had been placed additionally (72%). At the time of catheter removal, three patients (15%) stood out with a persistent stricture during the postoperative MCU. Three patients showed an extravasation on postoperative MCU.

### Success rate

Median follow-up was 35 months (IQR 16–45). According to our database analysis, telephone survey, and postal questionnaire, another surgery for urethral reconstruction was necessary in eight patients (33%) following discharge from the hospital after the initial surgeries due to the development of either a new fistula or a new stricture. Four (16%) reported a recurrence of stricture and six (24%) reported a recurrence of fistula. Median post operative uroflow improved in both groups (median Qmax 18 ml/s, Qave 7,9 ml flowtime 37 s, Volume 332 ml) with only minimal residual urine (mean 23,7 ml, SD 22.6, Table 2).

### Complications

The 30-day perioperative complication rate was low and all six complications (24%) noted were minor complications according to Clavien-Dindo Grade 1. Postoperative complications included asymptomatic bacteriuria before micturition cystourethrography (n = 3), persistent extravasation at the time of MCU (n = 2) and postoperative hypokalemia not requiring treatment (n = 1).

### Patient-reported outcomes

Overall, the complete extended postoperative PROM was returned by 13 of 25 patients, which translates into a response rate of 52% (50% of all stricture patients and 41% of all fistula patients). All patients reported an improvement in quality of life to some degree. No patient complained about incontinence or recurrent urinary tract infections.

### ICIQ-S

The overall ICIQ-S outcome score was high (mean sum score 20 out of 24, SD 2.8). The mean overall satisfaction with surgery item score was high (8.6 out of 10;SD 1.6). There were no significant differences between stricture and fistula only patients. Nine patients (82%) stated that they suffered no or only little pain perioperatively. Six patients (55%) stated the surgery outcome was better than expected, while five patients (46%) said the outcome was different or just as expected.

### Six-item LUTS score from the USS PROM

The mean LUTS total complaint sum score, derived from six items (ranging from 0 to 24 possible points), was 6.7 (SD 3.9). For various LUTS-related issues most patients reported no problems or only occasional difficulties. These included delayed bladder emptying (63%), urinary stream strength (82%), needing to strain to urinate (72%), problems initiating and stopping micturition (91%), and complete emptying of the bladder (81%). However, urinary dribbling was reported by 50% of patients. 61% of all patients stated that this could be improved via manual milking of the urethra.

## Discussion

The findings of our study offer critical insights into the complexities and challenges associated with urethral complications in transgender individuals undergoing transgender men gender-affirming surgery. The notably high recurrence rate of treatment failure, with 33% of patients requiring anew urethral reconstruction, underscores the importance of ongoing postoperative care and vigilance. Our findings align with previous studies, one reported a 47% success rate for EPA at a five-year follow-up [6], another a 73% success rates, leading to no further need of surgical intervention [13]. This emphasizes the crucial need for these procedures to be carried out by expert surgeons. Given the unprecedented growth in gender-affirming surgery, reconstructive urologists must be well-versed in surgical outcomes and complications to diagnose, consult, and ideally treat such patients [1]. However, there is a lack of evidence concerning treatment, making our study’s data essential for enhancing patient care within this particular patient group, as most studies focus on complications after primary phalloplasty. This study pioneers a comprehensive evaluation of both objective and subjective outcomes of urethral reconstruction, offering vital insights for clinical practice, particularly in preoperative patient counseling.

Despite the mentioned challenges, the substantial improvement in postoperative uroflow metrics indicates the effectiveness of the interventions, enhancing patients’ urinary function and quality of life. Especially the ability to void naturally is of high importance to trans men. One notable aspect of our study is the utilization of comprehensive PROMs to assess both functional outcomes and patient satisfaction. To our knowledge, no validated PROM tool has been employed for patients undergoing urethral reconstruction for fistulas or strictures, hence the data gathered with the USS PROM and ICIQ-S provide a nuanced understanding of patient experiences post-surgery. Despite the high recurrence rates, patients expressed significant satisfaction with the surgical outcome and urinary function, evident in the very low mean score on the six-item LUTS score and high ICIQ-S scores. As previously noted, we observed low complication rates, those predominantly being of low grade, another crucial aspect of preoperative patient counseling.

The utilization of diverse surgical approaches, including EPA, buccal mucosal grafts, and flap procedures, demonstrates the multifaceted nature of urethral reconstruction in transgender patients. Interestingly, we observed that nearly half of our patients were smokers, a well-documented risk factor for stricture recurrence [15]. The high prevalence of smoking within our patient cohort may contribute to the increased incidence of both fistula and stricture recurrence noted in our findings. Further comparative studies, albeit challenging given the patient population’s uniqueness and the rarity of these complications, are essential to establish best practices and evidence-based guidelines. However, we do feel that a surgeon should possess a repertoire of diverse surgical approaches, thereby ensuring preparedness to navigate a myriad of complex surgical scenarios. The high recurrence of urethral complications, even if performed in a specialized tertiary referral center dedicated to urethral reconstruction, highlights the need for ongoing research and refinement in surgical techniques.

Our study also highlights the importance of preoperative counseling and managing patient expectations realistically. While the overall satisfaction rates are high, the presence of complications and the need for recurrent surgeries emphasize the need for transparent communication between healthcare providers and patients. Addressing potential postoperative issues such as urinary dribbling, reported by 50% of patients as well as the possibility of additional surgeries should be a focal point in preoperative discussions, allowing patients to make informed decisions about their surgeries.

Thus, we feel that our findings provide essential data for clinical practice, particularly in preoperative patient counseling, aligning patient expectations with a realistic outlook. Despite these strengths, our study has several limitations. Primarily, being a retrospective observational study, we were unable to include preoperative PROM data and do a comparative analysis. Secondly, the sample size was insufficient for a robust comparison of different surgical techniques. Many other studies have faced similar difficulties and could not favor one surgical technique over another [16]. However, conducting a randomized high-quality study with adequate statistical power for such a comparison in this specific setting would be tremendously challenging. Thirdly, retrospective questionnaires are susceptible to selection bias, potentially leading to higher true recurrence rates. One might argue that the patient cohort is quite heterogeneous, as both patients with urethra fistula and stricture were included. Nevertheless, given the remarkably high rates of simultaneous occurrence we observed, a simultaneous analysis appears appropriate.

## Conclusions

In our cohort of trans men undergoing urethral reconstruction as treatment for urethral stricture and fistula, there was a high recurrence rate for both stricture and fistula. However, patient satisfaction with regards to the surgical outcome and improvement of quality of life was high. Prospective, multi-institutional collaborations should further evaluate the efficacy of different surgical approaches in this context.
